# Cancer Care Delivery Challenges Amidst Coronavirus Disease – 19 (COVID-19) Outbreak: Specific Precautions for Cancer Patients and Cancer Care Providers to Prevent Spread

**DOI:** 10.31557/APJCP.2020.21.3.569

**Published:** 2020-03

**Authors:** Abhishek Shankar, Deepak Saini, Shubham Roy, Alireza Mosavi Jarrahi, Abhijit Chakraborty, Sachidanand Jee Bharati, Farzad Taghizadeh-Hesary

**Affiliations:** 1 *Department of Radiation Oncology, Lady Hardinge Medical College, Directorate General of Health Services, Ministry of Health and Family Welfare, Government of India, *; 2 *Indian Society of Clinical Oncology, *; 3 *Sitaram Bhartia Institute of Science and Research, *; 6 *Department of Oncoanaesthesia & Palliative Medicine, Dr BR Ambedkar Institute Rotary Cancer Hospital, All India Institute of Medical Sciences, Delhi, India,*; 4 *Department of Social Medicine, Medical School, *; 7 *Department of Radiation Oncology, Shaheed Beheshti University of medical Sciences, Tehran, *; 7 *5Baylor College of Medicine, Houston, USA. *

**Keywords:** Cancer, coronavirus, COVID-19, patients

## Abstract

Coronavirus outbreak has affected thousands of people in at least 186 countries which has affected the cancer care delivery system apart from affecting the overall health system. Cancer patients are more susceptible to coronavirus infection than individuals without cancer as they are in an immunosuppressive state because of the malignancy and anticancer treatment. Oncologists should be more attentive to detect coronavirus infection early, as any type of advanced cancer is at much higher risk for unfavorable outcomes. Oncology communities must ensure that cancer patients should spend more time at home and less time out in the community. Oncologists and other health care professionals involved in cancer care have a critical opportunity to communicate to their patients to pass on right information regarding practice modifications in view of COVID-19 outbreaks. Countries must isolate, test, treat and trace to control the coronavirus pandemic. There is a paucity of information on novel coronavirus infection and its impact on cancer patients and cancer care providers. To date, there is no scientific guideline regarding management of cancer patients in a background of coronavirus outbreak.

## Introduction

*Overview of COVID – 19 outbreak*

Coronavirus disease 2019 (COVID-19), caused by SARS-CoV-2, is a novel coronavirus first detected in Wuhan, China in December, 2019. (CDC, 2020, March 21; WHO, 2020, January 12). Since then, around 12,784 people have died and more than 2,92,142 confirmed cases have been reported in at least 186 countries, territories or areas, according to the World Health Organization (WHO) by 21^st^ March, 2020. Europe has emerged as a new epicenter for COVID-19 with mortality and morbidity increasing daily. Highest number of deaths reported in a single day in Italy was 793 followed by Spain (285), Iran (123), France (112) on 21^st^ March 2020. Daily increase in number of cases and associated mortality has forced lockdown in various countries to check the spread of virus. Iran reported its single biggest rise in number of deaths from the coronavirus as another 147 people died, raising the country’s overall death toll to 1,135 out of 17,361 confirmed cases in Iran on 18^th ^March 2020 (WHO, 2020, March 21).

In the United States, the virus has now spread to all 50 states, infecting a total of 15219 people with death being reported in 201 patients by 21^st^ March, 2020. India has confirmed infection in 396 patients with mortality reported in 6 patients by 22^nd^ March 2020 (ICMR, 2020, March 22).

Indian authorities did not recommend to expand coronavirus testing initially, unlike most affected nations. In view of increasing COVID-19 positive cases, Indian Council of Medical Research (ICMR) revised testing guidelines on 20^th^ March, 2020 (ICMR, 2020, March 20) as this was well predicted that limited testing could leave many of the COVID-19 cases undetected and unnoticed in the world’s second-most populous country.

These numbers are changing every minute and incidence and mortality figures will be changed when this publication goes online. This alarming trend has made the whole world on lockdown mode. According to WHO, the new coronavirus so far has an average R0 (contagion metric) between 2 and 2.5, which means a person infected with COVID-19 can pass it on to more than two people, which is more than a seasonal flu (R0 at 1.3), Influenza A virus subtype H1N1 (R0 at 1.2 and 1.6) and Ebola (R0 at 1.6 to 2), but less than Severe Acute Respiratory Syndrome (SARS) (R0 at 4) and Middle East Respiratory Syndrome (MERS) (R0 at 2.5 and 7.2) (Zhao, 2020).

Self- initiated quarantine for 14 days by people with mild symptoms remains most important public health intervention, but testing of all suspected cases, symptomatic contacts of probable and confirmed cases, would still be required (WHO, 2020, February 11).

*Oncological determinants of COVID-19*

Cancer patients are more susceptible to COVID-19 in future, than individuals without cancer because of their systemic immunosuppressive state caused by the malignancy and anticancer treatments, such as chemotherapy, targeted therapy and immunotherapy. There will likely be more detailed studies of COVID-19 in future as currently a lot is still unknown about this disease and how it is spreading. In addition, there has not been any specific information regarding cancer patients and COVID-19. However, one study suggested a small percentage of patients had preexisting conditions including diabetes (6.4%), hypertension (12.8%), cardiovascular disease (3.7%), liver diseases (2.7%), malignancy (1.4%), and others (3.7%) (Cai et al., 2020).

Liang et al., (2020) reported a significantly higher incidence of severe events i.e. death or ICU admission requiring invasive ventilation among individuals with a cancer history than those without (39% vs. 8%; P = .0003), in 2,007 Chinese patients hospitalized with COVID-19. However, cancer history was present in only 18 patients, some of them were long term survivors while others had active disease. The smaller sample size and associated co-morbidities in cancer patients contributing to increase infection susceptibility, made the findings difficult to interpret. There is no guidelines on care of cancer patients with cancer types (e.g. breast, lung), therapy types (e.g. immunotherapy, targeted therapy), or subpopulation of cancer patients (e.g. children, elderly). 

The major risk for cancer patients is the inability to receive necessary medical services (both in terms of getting to hospital and provision of normal medical care once there) because of the outbreak. As per the Report of the WHO-China Joint Mission on COVID-19, cancer patients had an estimated 2-fold increased risk of COVID-19 than the general population. Patients with comorbid conditions had much higher rates: 13.2% for those with cardiovascular disease, 9.2% for diabetes, 8.4% for hypertension, 8.0% for chronic respiratory disease, and 7.6% for cancer (WHO, 2020, February 28).

In a study by Yu et al., (2020) a total of 1,524 cancer patients at tertiary cancer Institution of Wuhan University were reviewed in which cancer patients were estimated to have a 2-fold increased risk of COVID-19 in comparison to the general population. Study results suggested that hospital visit was a likely factor contributing to the increased incidence in cancer patients, but incidence of severe events was not higher than in the general population.

Oncologists should be attentive as patients with any type of advanced cancer are going to be at much higher risk for bad outcomes if they are infected with the coronavirus. The potential risk factors of older age, comorbidities and smoking history could help oncologists to identify patients with poor prognosis at an early stage (Zhou et al., 2020). Oncology communities must ensure that patients undergoing active cancer treatment tend to spend more time at home and less time out in the community. 

*Specific Precautions to prevent spread in cancer patients*

Cancer patients must be informed regarding signs and symptoms of COVID-19 and trained in social distancing along with other practices to maintain hygiene. There is no specific guideline regarding the use of mask in cancer patients and this needed to be explored.

A comprehensive evaluation should be performed for cancer patients when they are presenting with fever or other symptoms of infection as there is no specific guidance about COVID-19 testing in cancer patients. Cancer patients must continue their treatment unless they are in close contact with someone with COVID-19 or presenting with symptoms of cough, shortness of breath, breathing difficulties or high temperature. 

*Cancer patients planned for Surgery*

Elective surgeries for cancer patients can be rescheduled if possible. However, oncologists and patients must decide after looking into the potential harms of delaying needed cancer surgery. 

*Cancer patients undergoing radiotherapy or planned for it*

Oncologists should decide to postpone, discontinue or modify radiation therapy. Cancer patients have to report to the hospital after consultation with oncologists, keeping an individualize risk/benefit assessment in mind. 

*Cancer Patients on Immunosuppressive therapy or planned for it*

There is no direct evidence to support changing or withholding chemotherapy, targeted therapy, and immunotherapy in cancer patients. The clinical decision should be individualized taking cancer recurrence into account if therapy is delayed, modified or interrupted. Some patients might switch from intravenous chemotherapy to oral drugs or some may consider home infusion of chemotherapy to minimize the risk of infection. 

*Cancer patients planned for Stem Cell transplantation*

Planned allogeneic stem cell transplantation can be reasonably delayed in view of coronavirus outbreaks particularly in a situation where the disease is controlled with conventional treatment. Visitors must be restricted to meet post-transplant patients and may need to be screened for symptoms and potential exposure. 

*Role of prophylactic Antiviral therapy*

At this time, there is no evidence or published guidelines regarding the use of prophylactic antiviral therapy for COVID-19 in immunosuppressed patients. Oseltamivir is not known to be effective in the treatment of COVID-19. Many clinical trials are ongoing on the use of potential antiviral medications (e.g. chloroquine, remdesivir, lopinavir, and favipiravir). However, to date, none of these trials have been specified to cancer patients and all have been in patients with suspected or confirmed infection, and not in the prophylactic setting.

*Recommendation regarding cancer screening*

Patients are advised to postpone their planned cancer screening procedures for some time in which they are required to visit cancer centers. 

*Cancer patients on follow up*

Cancer patients on regular follow up can avoid hospital visits. Patients must contact Cancer Care Providers in case of contact history with coronavirus or having symptoms. 

*COVID-19 and Cancer Care Providers*

Cancer Care Providers i.e. doctors, nurses, technicians, caregivers, and all other allied professionals are at increased risk for coronavirus infection as chances of acquiring infection at workplace are high. At least 2,629 health workers have been infected and 13 doctors died of coronavirus since the onset of the outbreak in February, representing 8.3 percent of total cases in Italy by 19^th^ March 2020. Thus, cancer care providers must report to the authorities, in case of dry cough, fever, sore throat or in case of contact with someone with COVID-19. They need to be tested for COVID-19, followed by quarantine for 14 days or admission and treatment depending on the test results. 

Feeling under pressure and working under extreme circumstances are likely to be experienced by cancer care providers on many occasions. Extra work hours and sleepless nights in view of a large number of patients reporting to the hospital have become part of cancer care professionals. Stress and the panic associated with the current situation will be more evident in the near future, so managing mental health and psychosocial wellbeing during this time is as important as managing physical health.

*General Protection steps for cancer patients and caregivers*

• Handwashing with soap and water or alcohol based hand rub 

• Cover your mouth with a tissue while coughing and sneezing, dispose of the tissue, hand washing

• Avoid touching face with unclean hands

• Clean and disinfect frequently touched objects and surface 

• Avoid close contact with people – keep more than 1 meter distance between you and others

• Avoid crowded spaces, especially indoors.

• Avoid contact with anyone who is ill with a cough, fever or difficulty in breathing

• Do not share objects that touch your mouth – for example, bottles, cups.

• Do not shake hands.

• Practice social distancing

*General Protection steps for cancer care providers (WHO, 2020, March 20)*

Cancer Care providers should follow standard precautions like hand and respiratory hygiene, safe waste management, sterilization of equipments, etc. 

Ensure all patients should cover their mouth and nose if they cough or sneeze.

Arrange separate rooms for admission of suspected COVID-19 cancer patients or keep at least 1-meter distance between beds in ward admissions. 

Cancer care providers should wear clean sterile, protective personal equipment.

Limit the use of aerosol generator procedures for cancer patients like intubations.

Use of screening questionnaire for the detection of suspected coronavirus cases.

Perform hand hygiene after visiting every patient.

*Myths, misinformation and correlating facts*

Oncologists and other health care professionals involved in cancer care have a critical opportunity to communicate their patients to pass on the right information regarding practice modifications in view of COVID-19 outbreaks. But, a lot of information, shared on social media platforms, about coronavirus infection, its prevention and treatment are not supported by any scientific evidence. WHO has published an advisory for the public for myths and misinformation about COVID-19.(WHO, 2020) Some of the myths floating around are tabulated below so that cancer patients should not panic over.

*Adopt WHO Isolate, Test, Treat, Trace policy*

To suppress and control the epidemic, countries must isolate, test, treat and trace. If this is not adopted, transmission chains can continue at a low level and then resurface once physical distancing measures are lifted (WHO, 2020, March 18).

Every community needs to perform many more hard tasks encountering challenges and converting it into an opportunity. We must think, plan and perform professionally. Now and then, we need to self-assess our interventions, whether they are relevant in the changing environment. We need not confine ourselves in providing temporary remedies but strive for ensuring sustainable solutions. It is time for everyone to think globally and act locally to ensure that their intervention is relevant, appropriate, latest, and right in action.

**Table:** Extra Protection for Cancer Patients

Do’s
Tell visitors not to call if they have any symptoms of coronavirus to avoid unnecessary panic
Meet people in a well-ventilated room or outdoors.
Ask visitors to wash their hands properly.
Ask visitors to keep a space of at least 2 meters between you and them.
Make a joint plan with family, friends, and neighbors for the support you need now, or if you become unwell.
Refill your prescription medications and have over-the-counter medicines and supplies, for example, tissues and a thermometer
Keep physically active, if possible.
Don’t’s
Do not have any more than 2 visitors at a time to your home.
Do not shake hands with visitors.
Do not isolate yourself from friends and family.

Note: Extra protection is required for cancer patients who are older than 75 years but can be followed by all cancer patients on treatment

## References

[B1] Cai Q, Huang D, Ou P (2020). COVID-19 in a Designated Infectious Diseases Hospital Outside Hubei Province,China. Preprint. Posted online February.

[B2] Centers for Disease Control and Prevntion (CDC) Coronavirus disease 2019 (COVID-19) situation summary.

[B3] Indian Council of Medical Research (ICMR) Revised Strategy of COVID19 testing in India.

[B4] Indian Council of Medical Research (ICMR) SARS-CoV-2(COVID-19)Testing: Status Update.

[B5] Liang W, Guan W, Chen R (2020). Cancer patients in SARS-CoV-2 infection: a nationwide analysis in China. Lancet Oncol.

[B6] World Health Organization (WHO) (2020). Coronavirus disease (COVID-19) advice for the public: Myth busters.

[B7] World Health Organization (WHO) Novel coronavirus – China.

[B8] World Health Organization (WHO) Key considerations for repatriation and quarantine of travellers in relation to outbreak of novel coronavirus 2019-nCoV.

[B9] World Health Organization (WHO) Report of the WHO-China Joint Mission on Coronavirus Disease 2019 (COVID-19).

[B10] World Health Organization (WHO) WHO Director-General’s opening remarks at the media briefing on COVID-19.

[B11] World Health Organization (WHO) Infection prevention and control during health care when COVID-19 is suspected.

[B12] World Health Organization (WHO) Novel Coronavirus (COVID-19) Situation.

[B13] Yu J, Ouyang W, Chua MLK (2020). SARS-CoV-2 transmission in cancer patients of a tertiary hospital in Wuhan. Preprint. Posted online February.

[B14] Zhao S, Lin Q, Ran J (2020). Preliminary estimation of the basic reproduction number of novel coronavirus (2019-nCoV) in China, from 2019 to 2020: A data-driven analysis in the early phase of the outbreak. Int J Infect Dis.

[B15] Zhou F, Yu T, Du R (2020). Clinical course and risk factors for mortality of adult inpatients with COVID-19 in Wuhan, China: a retrospective cohort study. Lancet.

